# Socioeconomic Status, Health Behaviors, and Oral Health of Older Adults In China

**DOI:** 10.1093/geroni/igaa057.3047

**Published:** 2020-12-16

**Authors:** Yaolin Pei, Xi Chen, Michele Saunders

## Abstract

Many Chinese older adults suffer from oral health diseases and problems due to low oral health literacy, limited dental coverage and lack of dental care services for this segment of the population in China. However, so few studies have been conducted to examine social and behavior factors related to oral health among Chinese older adults. This symposium examines how socioeconomic status (SES) and health behaviors are associated with oral health among Chinese older adults. The first paper used the Nanjing Centenarians Study to examine the association between health behaviors and oral health among Chinese centenarians. The results showed that health behaviors were associated with self-rated oral health and edentulism. Using the Chinese Longitudinal Healthy Longevity Survey, the second one employed an ‘after death’ approach to examine risk factors for orofacial pain symptoms at the end of life among Chinese older adults. The third paper investigated the association between SES and tooth loss among middle-aged and older adults in ten cities of China. SES played a stronger role in tooth retention for non-migrants and migrants with high education vs those migrants with low education. The last paper examined the association between health behaviors and retention of teeth among Chinese older adults using data from the Chinese 4th National Oral health Survey. This symposium provides empirical evidence on the current status of oral health and health behaviors at the national level, and also suggests that is critical to improve oral health education and access to dental care.

